# Identification of the main allergen sensitizers in an Iran asthmatic population by molecular diagnosis

**DOI:** 10.1186/1710-1492-10-41

**Published:** 2014-08-05

**Authors:** Fardis Teifoori, Masoomeh Shams-Ghahfarokhi, Idoia Postigo, Mehdi Razzaghi-Abyaneh, Ali Eslamifar, Antonio Gutiérrez, Ester Suñén, Jorge Martínez

**Affiliations:** 1 Department of Mycology, Faculty of Medical Sciences, Tarbiat Modares University, Tehran 14115-331, Iran; 2 Laboratory of Parasitology and Allergy, Center for Research Lascaray Ikergunea, University of the Basque Country, Pº Universidad, 7, 01006 Vitoria, Spain; 3 Department of Immunology, Microbiology and Parasitology, Faculty of Pharmacy, University of the Basque Country, Pº Universidad, 7, 01006 Vitoria, Spain; 4 Department of Mycology, Pasteur Institute of Iran, Tehran 13164, Iran; 5 Clinical Research Department, Pasteur Institute of Iran, Tehran 13164, Iran

**Keywords:** Allergy, Atopy, Specific IgE, Component resolved diagnosis, Asthma, Risk factor, Pollen, Mould, Cockroach, Protein, Allergen

## Abstract

**Background:**

There has been a significant growth in the prevalence of allergy, mainly associated to IgE-mediated disorders such as asthma and rhinitis. The identification of atopy in asthmatic patients through the measurement of specific IgE can help to identify risk factors that cause asthmatic symptoms in patients. The development and use of individualized allergen-based tests by the Component Resolved Diagnosis has been a crucial advance in the accurate diagnosis and control of allergic patients. The objective of this work was to assess the usefulness of molecular diagnosis to identify environmental allergens as possible factors influencing the development and manifestation of asthma in a group of asthmatic patients from Iran.

**Methods:**

Studied population: 202 adult asthmatic patients treated at the Loghman Hakim Hospital and Pasteur Institute of Teheran (Iran) from 2011 to 2012. Specific IgE determined by the ImmunoCAP system were used to both evaluate the patients’ atopic condition and the molecules involved in the allergic sensitization. SDS-PAGE IgE-immunoblotting associated with mass spectrometry was carried out to study the cockroach IgE-binding sensitizing proteins.

**Results:**

Forty-five percent of all patients could be considered atopic individuals. Eighty-two percent of atopic patients were sensitized to pollen allergens. The *Salsola kali* (Sal k 1) and the *Phleum pratense* (rPhl p 1 and/or rPhl p 5) major allergens were the most common sensitizers among pollens (71% and 18%, respectively). Thirty-five percent of the atopic population was sensitized to cockroach. Four different allergens, including a previously unknown alpha-amylase, were identified in the cockroach extract. No significant associations could be demonstrated between the severity of asthma and the specific IgE levels in the atopic population. Statistical analysis identified the Sal k 1 as the main protein allergen influencing the development and expression of asthma in the studied population.

**Conclusions:**

Pollen and cockroach were the most relevant allergen sources in the asthmatic population. The *Salsola kali* major allergen was the main cause for sensitization in the atopic patients suffering asthma. Using the Component Resolved Diagnosis, it was possible to identify a new *Blattella germanica* cockroach allergen (*Blattella* alpha amylase 53 kDa) that could sensitize a relevant percentage of this population.

## Background

The World Allergy Organization defines the term atopy as a personal and/or familiar tendency to become sensitized and produce IgE antibodies in response to ordinary exposure to allergens, most frequently proteins. Common allergens associated with atopy are inhaled and food proteins [1]. There has been a significant exponential growth in the prevalence of atopy, including IgE-mediated allergic diseases such as asthma and rhinitis. It is estimated that 30% of the world population is now affected by one or more allergic conditions and is capable of developing specific IgE antibodies to different allergenic proteins from various sources, including pollens, molds, dust mites, insects, epithelia and foods [2].

Asthma is a worldwide problem, with an estimated 300 million affected individuals. The global prevalence of asthma ranges from 1-18% of the population, depending on the country. Indoor and outdoor allergens are important environmental factors that influence the development and expression of asthma. The identification of atopy through specific IgE determination can help to identify the triggers able to develop symptoms of allergic asthma in atopic patients [3]. Geographical variation in the prevalence of sensitization to common aeroallergens is commonly observed across different geographical sources [4] and several studies have reported on the aeroallergen sensitivity of Iranian patients with asthma or rhinitis [5-7] demonstrating significant variation in sensitization sources depending on the report. Despite the different localizations of these studies, all results agree that pollen sensitization is the most prevalent and that Chenopodiaceae, grass and sycamore are the most important allergenic sources. The arthropods most commonly associated with sensitization were house dust mite and cockroach, but their prevalence was very different according to their location.

The development and use of individualized native and/or recombinant allergen-based tests in the routine diagnosis of allergic diseases have been crucial advances in the accurate diagnosis and control of allergic patients [8]. This advance enables the progression from mere taxonomic diagnostic approximations to the molecular classification of allergenic substances [9] and, thus, from individualized allergens to clinical decisions [10]. Component Resolved Diagnosis (CRD) has been shown to be the most accurate methodology for the molecular diagnosis of allergy [3], and the use of this methodology would redefine concepts such as major allergens, cross-reactivity or primary sensitization [11-13]. The proteomics underlying CRD enables a more complete description of the molecular allergen panel, independent of the availability or knowledge of the allergen molecules involved [14].

With this in mind, the objective of this work was to demonstrate which environmental allergenic proteins could be the possible triggers of asthma in a population of atopic individuals suffering asthma symptoms in the north of Iran.

To the best of our knowledge, this is the first report to demonstrate the prevalence of aeroallergens in asthmatic patients from Iran evaluated by CRD.

## Methods

### Study population

Two hundred and two adult patients (aged 18–83 years, mean = 41 years) treated at the Loghman Hakim Hospital and Pasteur Institute of Teheran (Iran) from 2011 to 2012 were included in this study.

All these patients were diagnosed with asthma following the Global Initiative for Asthma criteria [15]. Respiratory symptoms (cough, dyspnea or chest tightness), spirometry measurements with and without bronchodilator, and methacholine tests (in normal pulmonary function test cases) were performed. Only those patients who were positive for methacholine or pulmonary function tests were selected for inclusion in the study. Oral informed and written consent for participating in this study was obtained from the patients. The study was approval by the Ethics Committee of the Loghman Hakim Hospital. The sex distribution was 40.1% (81) male and 59.9% (121) female.

### Atopy study

Atopy for all subjects was analyzed using the ImmunoCAP Phadiatop (ThermoFisher Sci. USA) assay [16], which is a solid-phase immunoassay for serum specific IgE against a balanced mixture of relevant inhaled and food allergens. The recommendations of the manufacturer’s protocol were strictly followed. Phadiatop ImmunoCAP results are displayed as qualitative positive or negative responses. Because *Salsola* allergen is not included in the ImmunoCAP Phadiatop, specific IgE to this allergen (Sal k 1) was measured for all subjects separately in an ImmunoCAP-specific IgE assay (ThermoFisher Sci).

### Specific IgE quantification

Specific IgE to allergens was quantified by the ImmunoCAP-specific IgE assay (ThermoFisher Sci. USA) following the recommendations of the manufacturer’s protocol. ImmunoCAP-specific IgE results are expressed in kU/L. Values ≤ 0.1 kU/L were considered negative results.

Specific IgE to allergen extracts from cockroach *(Blattella germanica)* was used because the difficulty in obtaining commercially available cockroach allergens.

### Component resolved diagnosis

Quantitative CRD was performed in the atopic subjects using ImmunoCAP technology (ThermoFisher Sci. USA) following the manufacturer’s instructions. The results are expressed in kU/L, and values ≤0.1 kU/L were considered negative results. The following allergens were used: rDer p 1, rDer p 2, rDer p 10, rPen a 1, rPhl p 1, rPhl p 5, rPhl p 7, rPhl p 12, nOle e 1, nSal k 1, nArt v 1, rFel d 1, rAlt a 1, rAsp f 1, r Asp f 6 and *Aspergillus oryzae* alpha amylase.

### Identification of *Blattella germanica* allergens

#### Cockroach extract

Cockroach crude extract was obtained from Bial Laboratories (Bial-Aristegui, Bilbao, Spain).

#### SDS-PAGE-IgE-Immunoblotting

Sodium dodecyl sulphate-polyacrylamide gel electrophoresis (SDS-PAGE) was performed on a 4% stacking gel and 12.5% resolving gel according to the method of Laemmli [17]. After protein separation, gels were either fixed and stained for crude protein using Coomassie Brilliant Blue R-250 or transferred to a polyvinylidene fluoride (PVDF) membrane [18]. The PVDF membrane was incubated overnight with the patient’s sera at 4°C. Bound IgE antibodies were detected using HRP-conjugated goat anti-human IgE and an ECL-Western blotting kit (Amersham ECL Plus Western Blotting Detection System, GE Healthcare UK Ltd, Buckinghamshire, UK) [19].

### Mass spectrometry and database searching

The IgE-binding bands revealed by immunoblotting were sent to the Proteomic Unit of Carlos III National Centre for Cardiovascular Disease Research Foundation (Madrid, Spain) for identification by MALDITOF-MS/MS. MALDI-MS and MS/MS data were combined using the BioTools programme (Bruker Daltonics, Billerica, MA, USA) to search protein databases (NCBInr; ~4.8 × 10^6^ entries; National Centre for Biotechnology Information, Bethesda, MD, USA; SwissProt; ~2.6 × 10^5^ entries; Swiss Institute for Bioinformatics, Switzerland) using Mascot software (Matrix Science, London, UK). MALDI-MS/MS spectra and database search results were manually inspected in detail using the above programs as well as homemade software and the Structural Database of Allergenic Proteins. The amino acid sequence was assessed by searching the NCBInr database with fragment ion masses from the precursor ion at m/z = 2710.286. Protein scores greater than 81 were considered significant (P < 0.5).

According to the Structural Database of Allergenic Protein (SDAP), possible cross-reactivity between a query protein and a known allergen has to be considered when there is an identification of six contiguous amino acids between the query sequence and any allergen [20]. The alpha-amylase amino acid sequence was assessed by searching the SDAP in this manner, following the database instructions.

### Statistical analysis

The data obtained were analyzed with GraphPad Prism 4.0 (La Jolla, CA. USA). Data are expressed as the mean and 95% confidence interval (CI). Means were compared using ANOVA. P values <0.05 were considered statistically significant. Logistic regression (SPSS Statistics 21. IBM) was used to assess the association between the quantification of major allergen-specific IgE and the severity of asthma.

## Results

Ninety-two subjects (45%) from the total evaluated sample (202 asthmatic patients) tested positive for Phadiatop and specific IgE to *Salsola kali*. All these individuals were considered atopic. Among the 202 patients studied, 57% presented with severe asthma, 36% with moderate asthma and 7% with mild asthma. The distribution of asthma severity among the atopic patients within the sample was 49% for severe asthma, 38% for moderate asthma and 13% for mild asthma. No statistically significant differences were found between the asthmatic population and the asthmatic population with atopy (p > 0.05).

Table 1 displays the results of the allergy diagnosis based on the CRD concept. Eighty- two percent of atopic patients were sensitized to pollen allergens, with *Salsola kali* major allergen (Sal k 1) as the most frequent allergenic source, which was able to sensitize 71% of the atopic subjects and was associated with higher specific IgE values (26.7 ± 21.0 kU/L). Taking into account the prevalence of sensitization to each allergen in the atopic population, the statistical analysis demonstrated that Sal k 1 was the primary sensitizing allergen in the asthmatic population, showing significant differences when compared with the remaining allergens studied (p ≤ 0.0001).

**Table 1:** Allergy diagnosis based on the allergen components

**Allergen**	**Mean sIgE (kU/L)**	**SD**	**Percentage (%)**
*Sal k* 1	26.7	18.1	71
*Phl p*1+ 5	7.64	10.8	18
*Fel d* 1	6.43	5.8	10.8
*Ole e* 1	2.9	3.3	7.6
*Art v* 1	1.4	1.3	7.6

Mean specific IgE values, standard deviation (SD) and percentage (%) of sensitized patients to each allergen in the atopic population (n = 92).

Thirty-five percent of the atopic subjects had anti-cockroach IgE responses, but the level of specific IgE was lower (2.8 kU/L ± 7). Major allergens from grass pollen (Phl p 1 + 5) showed a sensitization frequency of 18% and a specific IgE mean value of 7.64 ± 10.8 kU/L. Eighty-eight atopic patients (95%) were sensitized to pollens and/or cockroach allergens.

Although significant specific IgE values were discovered for other major allergens, such as Fel d 1 (6.43 kU/L ± 5.8), Ole e 1 (2.9 kU/L ± 3.3) and Art v 1 (1.4 kU/L ± 1.3), the sensitization rates were less than 15%. The remaining sensitizing allergen components (Alt a 1, Asp f 1, Asp f 6, Phl p 7 + 12, Der p 1, Der p 2, and tropomyosin) showed no significant specific IgE values (mean values ≤0.5 kU/L) or important frequencies (values less than 10%). Sensitization by cross-reactive allergens (pollen polcalcins/profilins and tropomyosin) reached 5.4% of atopic individuals (5 patients), with allergen-specific mean values ranging from 0.4 to 0.5 kU/L.

Table 2 depicts the results of a statistical analysis of the association between asthma severity and the levels of specific IgE in the atopic population. No significant associations were observed.

**Table 2:** Statistic analysis of the association between the severity of asthma and the level of specific IgE to the individual allergens in the atopic population

**Allergen**	**Odds ratio**	**P value**	**% 95 CI**
*Sal k* 1	1.071	0.516	0.870-1.319
Cockroach	0.992	0.969	0.673-1.462
*Phl p* 1 + 5	1.185	0.517	0.709-1.982
*Fel d* 1	0.934	0.824	0.511-1.707
*Ole e* 1	1.002	0.997	0.423-2.372

**Figure 1:**
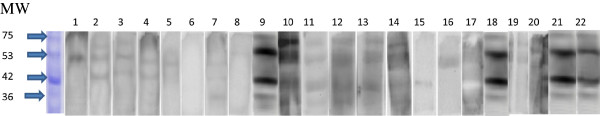
Allergogram of 22/33 atopic patients sensitized to cockroach crude extracts.

To identify which cockroach allergens were able to sensitize the atopic population, SDS-PAGE immunoblotting associated with MALDI-TOF-MS and MALDI-TOF-MS/MS were performed. Figure 1 shows the allergogram of the 22 from 33 atopic patients sensitized to cockroach crude extracts. Four IgE-binding components of 75.0, 53.0, 42.0 and 36.0 kDa molecular weight (MW), reactive in 63.6%, 86.4%, 72.7% and 54.5% of cockroach-sensitized patients, respectively, were revealed in this way. The prevalence rates of each uncovered cockroach allergen (75, 53, 42 and 36 kDa) in the total atopic population with asthma were 15%, 20%, 17% and 12%, respectively. Mass spectrometry analysis of the four bands allowed for the identification of only the 53 and 42 kDa proteins. Table 3 displays the results of mass spectrometry. These two proteins were identified as alpha-amylase and arginine-kinase, which have theoretical MWs of 57.5 and 40.1 kDa, respectively. No specific IgE to *Aspergillus oryzae* alpha-amylase was found in sera from 8 patients sensitized to *Blattella germanica* alpha-amylase (≤0.1 kU/L). A BLAST alignment of protein sequences of *Blattella germanica* alpha-amylase with *Aspergillus oryzae* alpha amylase showed a query cover and E-value of 50% and 4E-12, respectively. An SDAP search revealed that there was a match of at least of six contiguous amino acids between the alpha-amylase of *Blattella germanica* and the alpha-amylase of *Blomia tropicalis* (Blo t 4.0101)*, Dermatophagoides pteronyssinus* (Der p 4) and *Euroglyphus maynei* (Eur m 4), which are three described mite allergens.

**Table 3:** **Protein identification of *Blattella germanica***

	**SDS-PAGE protein 1**	**SDS-PAGE protein 2**
Calculated MW	53 kDa	42 kDa
Theorical MW	57.501 kDa	40.109 kDa
Protein description	Alpha amylase	Arginine kinase
Peptide sequence matched	r.saivhlfewkfadiadecerf.l	k.laasdsksllr.k
	k.gfagvqvspvhenviisspfrpwwer.y	k.hppkdwgdvdtlgnldpageyiistrvrcgrsmqgypfnpclteaqyk.e
	v.rncelvglhdlnqgsdyvr.g	k.gqfypltgmtk.e
	k.vnnlntdhgfpsgarpffyqevidlggeaihsteytgfgr.v	r.flqhanacr.f
	k.mavafmlaypygyp.r	k.tflvwcneedhlriismqmggdlgqvyrrlvtavndiekrvpfshddrlgf.l
	r.qifnmvgfrnavagtavsnwwdngdkqisfcr.g	
	k.gfvafndefnndlk.q	
Sequence covered	36.31%	43.5%
Mascot score	200	292
Mascot expect	3.3E-014	2.1E-023
NCBI accession number	gi|85002763	gi|86160922

Results of Mascot search.

## Discussion

Asthma is a common worldwide health problem with a high global prevalence ranging from 1-18% of the population depending on the country. Its association with elevated total serum IgE and with allergic sensitization to local aeroallergens has been well documented [21,22]. Indoor and outdoor allergens are important environmental factors influencing the development and expression of asthma, and the measurement of specific IgE can better define an individual’s atopic condition [23].

Several studies have demonstrated that the prevalence of atopy is varied among asthmatic patients. Arbes *et al.*[24] have reported that approximately 50% of the current asthma cases in the US population are attributable to atopy, whereas Sunyer *et al.*[25] have found that the overall attributable fraction of asthma symptoms caused by atopy in Europe is 30%, although varying widely between centres, from 4% to 61%. In our study, the prevalence of atopy in the asthmatic population was of 45%. Despite the prevalence, the variability was wide, and considering the possible role of environmental allergens as a trigger of asthma development, the attributable fraction of asthma symptoms related to atopy in the population studied in this work agreed with the results found by the European studies. Vernon *et al*. [26] have reported that the most cited triggers of asthma are very similar across countries/regions and included allergens (particularly pollens, moulds, dust and pet dander), tobacco, exercise, air pollutants/particulates, weather patterns/changes and respiratory diseases. They concluded that a global checklist should be used in research and clinical practice. Despite this similarity in asthma triggers, the geographical differences in the sensitization to environmental aeroallergens [27] and in the allergen sources (fungi, plants and animals) distributed across each region [28] make the study of allergen distribution necessary in each area.

Component Resolved Diagnosis [11-13] has been shown to be a more accurate methodology for the diagnosis of allergy, allowing for a very fine definition of the causes of allergic sensitization from a molecular perspective. Proteomics appears to provide a crucial added value in the molecular diagnosis of allergy, especially when individualized allergens are not available or when identifying novel allergens [14]. Sensitization to specific allergens and/or to panallergens or cross-reactive allergens offers an accurate evaluation of the diagnosis according to the concepts of cross-reactivity, co-sensitisation and polysensitisation. Taking into account the abovementioned concepts and in accordance with the sample studied and the geographical location of the individuals included in this work, we studied a sensitization panel of individual allergen components belonging to allergenic sources distributed throughout the North-Central part of Iran that contribute to the allergen sensitization in these patients. Major allergens implicated in the sensitization of the studied asthmatic population were the *Salsola kali* major allergen Sal k 1 (71% prevalence), the grass major allergen groups 1 and 5 (18% prevalence), the cat major allergen Fel d 1 (10%), the *Artemisia vulgaris* major allergen Art v 1 (7.6% prevalence) and the *Olea europaea* major allergen Ole e 1 (7.6% prevalence). Only five asthmatic patients were sensitized to pollen cross-reactive allergens (profilins or polcalcins) and the panallergen tropomyosin, with very low specific IgE values (≤0.5 kU/L), suggesting that the phenomenon of cross-reactivity is of little relevance in these patients.

The prevalence of sensitization to *Blatella germanica* as an allergenic source was relevant (35%) although the level of specific IgE was lower compared with the other sources described above (Sal k1, Phl p 1 + 5, Fel d 1 and Ole e 1). From the immunoblotting results, 4 different IgE-binding components (75.0, 53.0, 42.0 and 36.0 kDa) from cockroach were identified, with sensitivity prevalence rates of 15%, 20%, 17% and 12%, in our atopic patient population, respectively.

According to WHO guidelines [29], cross-reactivity between an expressed protein and a known allergen has to be considered in cases in which there are at least six contiguous identical amino acids or when a segment of at least 80 residues shares more than 35% identity at the amino acid level. Using these guidelines, cockroach arginine kinase (42 kDa) and cockroach alpha-amylase (53 kDa) were identified. Allergenic molecules homologous to the identified IgE-binding proteins were used to search the Allergome and IUIS *Allergen* Nomenclature databases (http://www.allergome.org, http://www. allergen.org), resulting in the identification of arginine kinase as the Bla t 9 cockroach allergen [30]. No homologue to the alpha-amylase cockroach allergen has been previously described; therefore, a novel major allergen from cockroach sensitizing 86.4% of cockroach-sensitized patients was identified. To test for cross-reactivity with this novel cockroach allergen, sera from reactive individuals were tested for their specific IgE reaction to an available alpha-amylase (*Aspergillus oryzae*) allergen and by searching the SDAP to demonstrate that the fragment identified as a *Blattella germanica* alpha-amylase could be included in the allergen group belonging to the alpha-amylase superfamily because of its similarity (six contiguous amino acids) with other alpha-amylase allergens. We found no cross-reactivity with *Aspergillus oryzae* alpha amylase, and BLAST alignments of cockroach alpha-amylase with other reported allergenic alpha-amylases [31] showed limited homology. The SDAP revealed that alpha-amylase allergens from *Blomia tropicalis* (Blo t 4.0101)*, Dermatophagoides pteronyssinus* (Der p 4) and *Euroglyphus maynei* (Eur m 4) have the defined requirements for cross-reactivity with the *Blattella germanica* alpha-amylase. However, the lack of IgE reactivity against *Dermatophagoides* allergens found in this report suggests that there is little cross-reactivity between cockroach alpha-amylase and alpha-amylase from mites in this patient population.

The statistical analysis between the prevalence of sensitization by each allergen in the atopic population and the level of sensitization as indicated by specific IgE, suggested that Sal k 1 was the main protein allergen influencing the development and expression of asthma in this population. Several studies have reported on the aeroallergen sensitivity of Iranian patients with asthma or rhinitis [5-7,32], and they found different prevalence rates among atopic subjects and different percentages of allergen sensitization. However, all of these studies agree that pollen sensitization is the most prevalent and that Chenopodiaceae, grass and sycamore are the most important allergenic sources. In the present work, *Salsola kali* and grasses were also found to be the most important allergens, although no sensitizations to major allergens from sycamore were discovered. The prevalence of pollen sensitization found in this work was 37% of the total asthmatic population and 82% of the allergen-sensitized population. The aforementioned authors [5-7,32] reported that indoor allergens were the second most common cause of sensitization after pollens. Mite sensitization ranged from 43% to 18%, and cockroach sensitization ranged from 11% to 29% [33,34]. In our study, however, no sensitization to mites was demonstrated, although the prevalence of cockroach sensitization was 16% among the total asthmatic population and 35% among the allergen-sensitized population. Moghtaderi *et al.*[35] have reported a mold sensitization prevalence of 11%, with *Aspergillus* as the most prevalent source, followed by *Cladosporium, Alternaria, Penicillium* and *Rhizopus.* In this work we did not find significant sensitization to major allergens from the fungi *Aspergillu*s or *Alternaria.*

## Conclusions

Pollen and cockroach were the more relevant allergen sources in the asthmatic population studied herein, with the *Salsola kali* major allergen serving as the main cause for sensitization in the atopic patients suffering asthma. A new *Blattella germanica* cockroach allergen (*Blattella* alpha amylase 53 kDa) has been identified as a major allergen of *Blattella*.

To the best of our knowledge, this is the first study examining a panel of sensitizing allergens in a population of asthmatic patients living in Iran for molecular diagnostic purposes. Using this methodology, it was possible to identify new allergens from cockroach that could sensitize a relevant percentage of this population.

These results indicate that cross-reactivity between pollens or arthropods do not play any significant role in the sensitization phenomenon of these patients.

## Abbreviations

CRD: Component resolved diagnosis; SDS-PAGE: Sodium dodecyl sulphate-polyacrylamide gel electrophoresis; PVDF: Polyvinylidene fluoride; SDAP: Structural database of allergenic proteins; NCBI: National Center for Biotechnology Information; BLAST: Basic local alignment search tool; ANOVA: Analysis of variance; US: United States; WHO: World Health Organization; IUIS: International Union of Immunological Societies.

## Competing interests

The authors declare that they have no competing interests.

## Authors’ contributions

FT: carried out the molecular diagnosis and drafted the manuscript. MS: participated in the study design and coordination and helped to draft the manuscript. IP: participated in the design of the study and coordination, helped to draft the manuscript and performed the statistical analysis. MR: participated in the study design and coordination and helped to draft the manuscript. AE: participated in the study design and coordination and helped to draft the manuscript. AG: participated in the sequence alignment and drafted the manuscript. ES: participated in the study design and coordination and helped to draft the manuscript. JM: conceived of the study, and participated in its design and coordination and helped to draft the manuscript. All authors read and approved the final manuscript.
